# The significance of multidisciplinary classifications based on transbronchial pathology in possible idiopathic interstitial pneumonias

**DOI:** 10.1097/MD.0000000000020930

**Published:** 2020-07-10

**Authors:** Weixue Wang, Jian Xu, Chunfang Liu, Ruie Feng, Junjun Zhao, Na Gao, Ling Jiang, Xiaolin Zhang, Xue Han, Lina Ren, Xiaohui Zhao, Yuan Liu

**Affiliations:** a Department of Respiratory; b Department of Anesthesiology; c Department of Pathology, Peking Union Medical College Hospital, Beijing, China.; d Department of Pathology, Dalian Municipal Central Hospital affiliated of Dalian Medical University, Dalian, China.

**Keywords:** bronchoscopy and interventional techniques, idiopathic interstitial pneumonias, interstitial lung diseases, transbronchial lung cryobiopsy, transbronchial pathology

## Abstract

Surgical lung biopsy is regarded as the golden standard for the diagnosis of idiopathic interstitial pneumonias (IIPs). Here, we attempted to show the diagnostic accuracy of multidisciplinary classifications based on transbronchial pathology including transbronchial lung cryobiopsy (TBLC) , bronchoalveolar lavage fluid (BALF) and endobronchial ultrasound-guided transbronchial needle aspiration biopsy (EBUS-TBNA).

Patients with suspected interstitial lung diseases admitted from June 1, 2016 to December 31, 2018 were involved. Patients with known causes of interstitial lung diseases and typical idiopathic pulmonary fibrosis diagnosed through clinical, radiological information were excluded. Patients with atypical idiopathic pulmonary fibrosis and possible IIPs accepted transbronchial pathological evaluation. Initial multidisciplinary diagnosis (MDD) classifications were made depending on clinical, radiological and transbronchial pathological information by a multidisciplinary team (MDT). The final MDD classifications were confirmed by subsequent therapeutic effects. All patients were followed up for at least 6 months.

A total of 70 patients were finally involved. The samples of lung parenchyma extracted through TBLC were enough for confirmation of pathological diagnoses in 68.6% (48/70) cases. Samples of 6 cases were extracted by EBUS-TBNA. Bacteriological diagnoses were positive in 1 case by BALF. Pathological diagnoses of 77.1% (54/70) cases were achieved through TBLC, EBUS-TBNA and BALF. During the follow up study, the pulmonary lesions of 60% patients were improved, 11.43% were relapsed when glucocorticoid was reduced to small dose or withdrawal, 14.29% were leveled off and 8.57% were progressed. The diagnoses of 4 patients with progressed clinical feature were revised. As a result, 94.3% initial MDD classifications based on transbronchial pathology were consistent with the final MDD, and the difference of diagnostic yield wasn’t significant between initial and final MDD (*Z* = −1.414, *P* = .157).

Classifications of IIPs based on transbronchial pathology were useful and quite agreed with final MDD.

## 1 Introduction

The idiopathic interstitial pneumonias (IIPs) comprise a number of clinicopathological entities, which are quite different as separate diseases. As a group, these diseases can be distinguished from other forms of diffuse parenchymal lung diseases (DPLD) through clinical methods including history collection, physical examination, chest radiology observation, laboratory tests, and pathological evaluation. Idiopathic indicates unknown causes and interstitial pneumonia refers to involvement of the lung parenchyma by varying combinations of fibrosis and inflammation. As American Thoracic Society/European Respiratory Society’ (ATS/ERS) new classifications,^[1]^ IIPs are now divided into major, rare and unclassifiable. The major IIPs are further divided into idiopathic pulmonary fibrosis (IPF), nonspecific interstitial pneumonia, respiratory bronchiolitis-associated interstitial lung disease (RB-ILD), desquamative interstitial pneumonia, cryptogenic organizing pneumonia, and acute interstitial pneumonia. Those associated with occupational or environmental exposures, and/or collagen vascular disease, granulomatous lung disorders are excluded.^[2]^ According to current guidelines,^[1,2,3]^ the diagnosis of IIPs requires a multidisciplinary discussion (MDD) with the reconciliation of clinical, radiological and histopathological information. MDD based on clinic–radiologic–pathologic information has been widely used as the diagnostic gold standard. Except typical IPF as the only IIPs for clinic-radiologic diagnosis,^[4,5]^ pathological evaluation is recommended for all others.^[6]^ Surgical lung biopsy (SLB) is recommended for suspected IIPs involving atypical IPF indicated by high resolution CT (HRCT) scans.^[6]^ Enough lung tissue can help pathologists to identify characteristic pattern and make the histopathological diagnosis. However, SLB which represents only a small sample of the whole lung and the minimal quantity of lung is necessary to guarantee the maximum morphological information.^[7]^ Recently, flexible cryoprobes have been used for peripheral lung parenchymal biopsy. Studies on the use of flexible cryoprobes for transbronchial lung cryobiopsy (TBLC) have shown the improvements in diagnostic yield, sample size, and architectural preservation of the biopsy specimens.^[8,9]^ Its’ adverse events were significantly lesser than SLB.^[10]^ Along with the progress of transbronchial intervention techniques, the more invasive diagnostic techniques including bronchoalveolar lavage (BAL), transbronchial forceps lung biopsy (TBLB), TBLC and endobronchial ultrasound-guided transbronchial needle aspiration biopsy (EBUS-TBNA) can be used for bacteriological, cytological and pathological evaluation.^[11]^ In this study, we attempt to show the diagnostic accuracy of MDD classifications based on transbronchial pathology including TBLC, BAL and EBUS-TBNA in IIPs.

## 2 Methods

### 2.1 Study population

We enrolled all new inpatients with suspected ILD detected by chest CT or HRCT scans at the respiratory department of Dalian Municipal Central Hospital affiliated of Dalian Medical University from June 1, 2016 to December 31, 2018. All patients completed medical history collection, physical examinations, serological examinations, pulmonary function tests and chest HRCT scans. The diagnoses of typical IPF, atypical IPF, or possible IIPs were was made by a multidisciplinary team (MDT) composed of rheumatologists, respiratory physicians, radiologists and pathologists. Patients with known causes of ILD such as occupational or environmental exposures and collagen vascular disease were excluded. Patients with atypical IPF and possible IIPs were finally eligible. Transbronchial pathological evaluation were performed in patients with atypical IPF and possible IIPs. Informed consents were signed before transbronchial examination from all patients.

### 2.2 Ethics

This study was approved by the Human Ethics Committee of Dalian Municipal Central Hospital.

### 2.3 Transbronchial examinations

Patients accepted arterial blood gases, electrocardiography, cardiac and pulmonary function test before operation. Transbronchial examination was prohibited in patients with forced vital capacity less than 50%, respiratory failure and cardiac insufficiency. All anticoagulation agents have been blocked for 5 days and replaced to low molecular heparin, including aspirin, clopidogrel or warfarin etc. Solids and liquids were withheld for 6 hours prior to the procedure. TBLC and BAL were performed in all cases by bronchoscopy (Olympus, BF-IT260, Japan) using a cryoprobe (ERBE ErbokryoCA, 1.9 mm diameter, Germany) under the guidance of radical endobronchial ultrasond (Olympus UM-S20-17S, 1.4 mm diameter, Japan). Patients accepted intravenous deep sedation and breath through mechanical ventilation by a size 4 laryngeal mask airway during operation. The target subsegment bronchus, where TBLC was performed, was the most affected pulmonary parenchymal areas matched chest HRCT images. Pathological specimens were sampled by TBLC at 1 to 3 target subsegments in each case and 1 to 3 specimens in each site. Bronchoalveolar lavage (BAL) was performed at bronchus of right middle lobe, left lingular segment or most affected pulmonary segment detected by chest HRCT. Bronchoalveolar lavage fluid (BALF) was collected for bacteriological and cytological examination. EBUS-TBNA was simultaneously conducted in patients with the enlarged mediastinal lymph nodes.

### 2.4 The complication evaluation

The bleeding complication which required intravenous hemostatic drug administration, cessation of transbronchial procedure or further intervention such as inflation of the bronchial blocker was defined as severe complication, and the bleeding which associated with cardiopulmonary instability, transfusion of packed RBCs or surgical intervention was defined as fatal complication.

### 2.5 The processes of MDD classification

Pathological diagnosis was made by 2 blinded pathologists discussing together. Initial MDD classifications were reconciled by MDT based on clinical, radiological and thransbronchial pathological information according to ATS/ERS classification.^[1]^ Appropriate treatments were carried out according to initial MDD classifications.

### 2.6 Final MDD classification

At least 6 months’ follow-up was conducted for all cases. The remission cases were that with disappeared symptoms and lesions on chest CT. The improved cases were that with alleviated symptoms and decreased lesions. The initial MDD classifications were confirmed as the final classifications if diseases were improved or remission. Further clinical, radiological and pathological evaluation were performed again if the disease progressed.

### 2.7 Statistical analysis

Statistical analyses were carried out using SPSS 22.0. Data was reported as the median and standard deviation for continuous variables, and rates and percentages for discrete variables. The diagnostic yield between the initial and final MDD classifications was compared using standard statistical approaches of Wilcoxon and McNemar nonparametric tests for categorical variables. The *P* value of <.05 was defined as a statistically significant difference.

## 3 Results

### 3.1 Clinical features

Finally, 70 eligible subjects were enrolled. Clinical features were presented in Table 1.

**Table 1:**
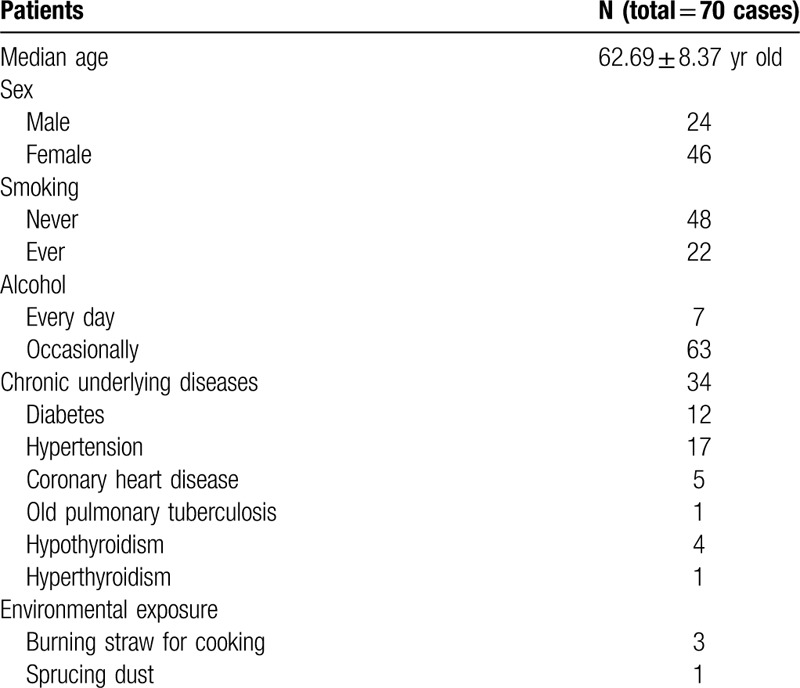
Clinical parameters of patients.

### 3.2 The result of tranbronchial pathology

TBLC were performed in all cases. TBLC was completed in 3 different subsegments in 10 cases, 2 subsegments in 56 cases and only 1 subsegment in 4 cases owing to severe bleeding. The rate of severe bleeding complication was 5.71% (4/70 cases). No fatal bleeding happened. The rate of pneumothorax was 4.29% (3/70 cases). Pneumothorax in the 3 cases was treated with drainage. No other complications occurred. One case with progressed and serious nonspecific interstitial pneumonia died after 2 weeks owing to severe condition before TBLC.

Whether or not the samples of lung parenchyma from TBLC being enough for pathological diagnosis was discussed by the MDT. The confirmed pathological diagnoses were made in 68.6% (48/70) cases, and 31.4% (22/70) cases didn’t get definitive pathological confirmation owing to inadequate samples, especially 2 cases involved only bronchus.

All cases accepted BAL examination. EBUS-TBNA were simultaneously conducted in 6 cases. Positive results were shown in Table 2. Pathological diagnoses were confirmed in 5 cases by EBUS-TBNA. A bacteriological diagnosis was gotten by BALF in 1 case. Confirmed pathological evaluations were made through TBLC, EBUS-TBNA and BALF. A total of 77.1% (54/70) cases got the definitive diagnosis based on transbronchial pathology.

**Table 2:**
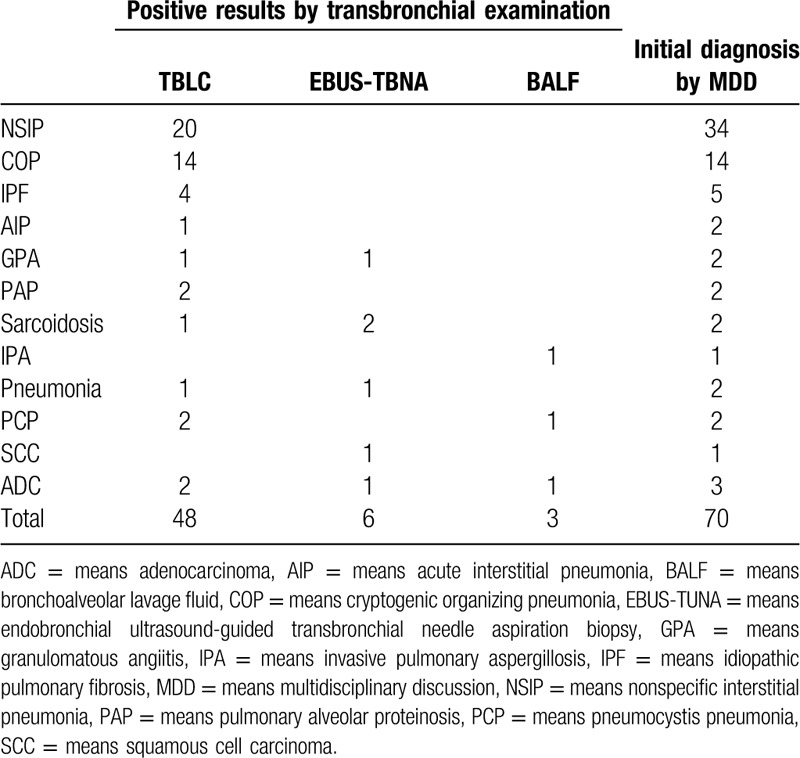
Diagnosis based on TBLC, EBUS-TUNA, BALF or MDD.

### 3.3 The accuracy of initial MDD

**Figure 1:**
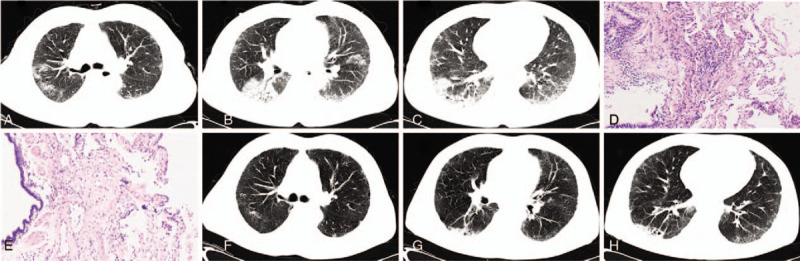
A case of NSIP was evaluated by initial MDD classifications. Bilateral ground glass opacity (GGO) was presented on the chest CT examined on May 5, 2017 (A, B, C). The fibrous proliferation in pulmonary interstitial and lymphocytes infiltration in alveolar septum were detected in the histopathological slices (HE stained × 100) based on TBLC at right upper lobe (E) and lower lobe (F). GGO was partially resolved at following chest CT on Jun 22, 2017 (G, H, I) after prednisone (1 mg/kg per day) treatment for more than 1 mo. MDD = multidisciplinary discussion, NSIP = nonspecific interstitial pneumonia.

**Figure 2:**
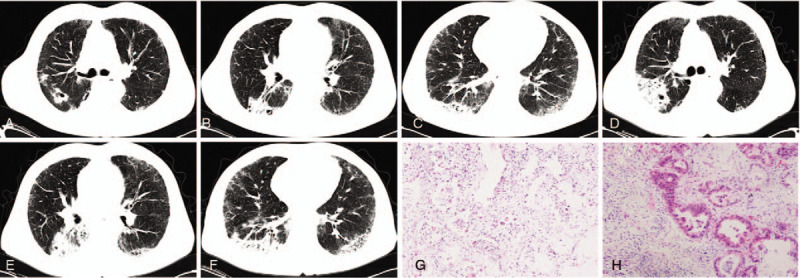
The same case in Figure 1. Some lesions progressed and consolidated at chest CT on Jun 7, 2018 (A, B, C) when prednisone was reduced to 15 mg per day, and the progression was much more obvious on Dec 27, 2018 (D, E, F). TBLC was performed once again, and the diagnosis of NSIP was modified as pulmonary adenocarcinoma in the histopathological slices (HE stained × 100) of right upper lobe (G) and lower lobe (H).

Initial MDD classifications were made depending on the combination of clinical, radiological and pathological information according to MDD (Table 3). Appropriate therapies were given after initial MDD classifications. The prognosis of patients were followed up for more than 6 months for all cases except 1 who died within 2 weeks. During the following-up, the pulmonary lesions of 60% (42/70) cases were improved, 11.43% (8/70) cases got remission but relapsed when glucocorticoid was reduced to small dose or withdrawal which had been diagnosed as cryptogenic organizing pneumonia by initial and final MDD, 14.29% (10/70) cases were steady, 8.57% (6/70) cases were progressed among which 1 had got the improvement about a year by taking prednison (Fig. 1) but gradually progressed after that (Fig. 2). Among the progressed cases, 6 of them were evaluated again based on the clinical, radiological and serological examination, and 3 of them accepted transbronchial pathological reexamination. As a result, the diagnoses of 4 cases were revised to systemic lupus erythematosus (1/4), anti-neutrophil cytoplasmic antibodies (ANCA) associated systemic vasculitis depending on serological examination (1/4) and pulmonary adenocarcinoma depending on TBLC reexamination (2/4) (Table 2). The diagnoses of 2 cases were as before because 1 case was died in 2 weeks and another was confirmed same diagnosis by a repeated TBLC. In addition, 5.7% (4/70) cases died of progressed IIPs including acute exacerbation in 1 case after 8 months’ improvement. Moreover, 5.7% (4/70) cases died of other diseases. The final MDD classification were also listed in Table 1. Among them, 94.3% (66/70) initial MDD classifications based on transbronchial pathology were consistent with the final. The difference wasn’t significant between initial and final MDD classifications (Z = −1.414, *P* = .157), the probability was 0.500 by McNemar Test.

**Table 3:**
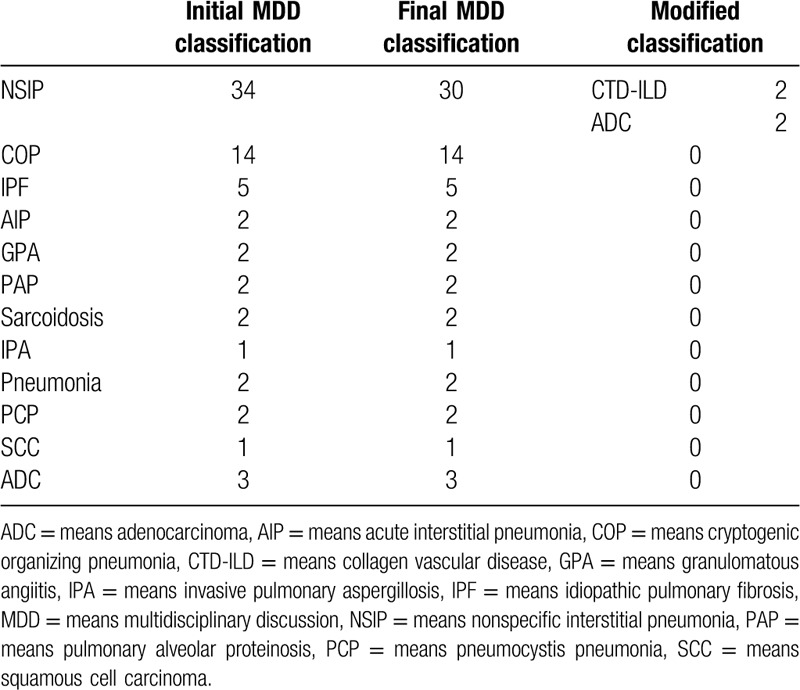
MDD classifications and following results (cases).

## 4 Discussion and conclusions

A broad range of non-neoplastic and non-infectious DPLD existed under the less specific umbrella term of ILD. This large group of diseases included those with histopathological findings identical to IIPs but with known causes, such as connective tissue diseases, respiratory exposures and those with unique clinical, radiological and pathological definitions, such as lymphangioleiomyomatosis, sarcoidosis, pulmonary langerhans cell histiocytosis and eosinophilic pneumonia etc. Some neoplastic or infectious processes masquerading as ILD were also commonly encountered. So, the pathological diagnosis only from pulmonary parenchyma wasn’t enough in some conditions. Microbiological and cytological information might also be needed in these cases. The progress of transbronchial techniques ensured all these procedure to be performed at the same examination. BALF was not always required in the assessment of the IIPs but to exclude infection or tumor.^[2,3]^ TBLB wasn’t used to diagnosing IIPs because traditional forceps yielded small biopsy samples with significant crush artifacts which were only sufficient for 20–30% overall diseases.^[6]^ Many studies indicated that the mean length and area of lung parenchymal tissues by TBLC were substantially larger than TBLB, and the specimens didn’t have crush artifacts.^[6,10,12–14]^ Samples obtained through TBLC contained peripheral structures of the secondary pulmonary lobules.^[8]^ There was a significant difference between TBLC and TBLB in terms of the percentage of sampled tissue containing open alveoli.^[10]^ The mean maximal diameter of the samples were around 9 mm.^[15,16]^ It was sufficient for histological diagnosis that the samples were larger than 5 mm in diameter as the expert's suggestion.^[17]^ Although SLB had been recommended as golden standard for pathological biopsies, the morbidity and mortality limited its clinic performance. A large dataset study showed the rate of in-hospital mortality following SLB for DPLD was 1.7%, and the rate of complication was 30% including postoperative pneumothorax, pneumonia, and respiratory failure.^[18]^ Bleeding complications occurred in 22% patients, and pneumothorax was 1.4%. The adverse events during TBLC were significantly lower than during SLB.^[6,11]^ Mortality due to adverse events was observed for 2.7% (SLB) and 0.3% (TBLC) of the patients.^[18]^ In our study, the rate of severe bleeding which hampered examination was 5.7%, and the rate of pneumothorax was 2.9%. The advantage of TBLC was more suitable for following and supervising the progressive diseases than SLB owing to less adverse events.

As the gold standard approach for the diagnosis of IIPs, MDD classifications needed to be reached by a multidisciplinary team comprised of expert pulmonologists, pathologists and radiologists after reviewing the available clinical, radiological and pathological data. Clinical and radiological information alone might lead to typical IPF but histological information were needed in other IIPs.^[6,19]^ Tomassetti^[20]^ indicated that the diagnostic confidence was significantly increased depending on the histopathological information from both TBLC and SLB in IPF compared with only clinical-radiological diagnosis. As for DPLD, Ravaglia^[21]^ compared diagnostic yield and showed that the diagnostic yield was 82.8% in TBLC vs 98.7% in SLB. Kropski^[16]^ and Poletti^[18]^ reported that pathological diagnostic pattern from TBLC was 80%. Ussavarungsi^[15]^ indicated that the definite MDD was yielded in 51% of subjects, and nonspecific histopathologic finding was in 49%. Hetzel indicated that the nondiagnostic rate was about 20% of cryobiopsies,^[17]^ the reasons included inadequate lung tissue, normal lung tissue or lung tissue with very minor and nonspecific pathology. In our study, the confident diagnostic yield based on transbronchial pathology in IIPs was 77.1%. Besides, 94.3% (66/70) initial MDD classifications based on transbronchial pathology was agreed with the final. As 5.7% initial MDD classification was modified through reevaluation and reexamination because of progressed pulmonary interstitial lesions after treatment, following-up the effect of therapy was essential for MDD classifications based on transbronchial pathology. MDD classifications of IIPs based on transbronchial pathology was useful and quite agreed with the final. The following up of therapeutic effect was also necessary for the confirmation of MDD classifications based on transbronchial pathology.

## Author contributions

Prof. JX and CL conceptualized the study and wrote the paper. Prof. RF and JZ gave their academic advice and paper check. WW, XH, NG, LJ, XZ, LR, XZ and YL collected cases, performed all tests and edited the figures.

**Conceptualization:** Chunfang Liu.

**Data curation:** Na Gao.

**Investigation:** Ling Jiang, Xiaolin Zhang, Xue Han, Lina Ren, Xiaohui Zhao, Yuan Liu.

**Methodology:** Ruie Feng, Junjun Zhao.

**Writing – original draft:** Weixue Wang, Jian Xu.
